# An epistemic community comes and goes? Local and national expressions of heart health promotion in Canada

**DOI:** 10.1186/1472-6963-9-35

**Published:** 2009-02-23

**Authors:** John Eyles, Kerry Robinson, Susan Elliott

**Affiliations:** 1 School of Geography and Earth Sciences, McMaster University, Hamilton, Ontario, Canada; 2 Public Health Agency of Canada, Ottawa, Ontario, Canada

## Abstract

**Background:**

The objective of this study is to examine the existence and shape of epistemic communities for (heart) health promotion at the international, national, provincial and regional levels in Canada. Epistemic community may be defined as a network of experts with an authoritative claim to policy relevant knowledge in their area of expertise.

**Methods:**

An interpretive policy analysis was employed using 60 documents (48 provincial, 8 national and 4 international) and 66 interviews (from 5 Canadian provinces). These data were entered into NUD*IST, a qualitative software analysis package, to assist in the development of codes and themes. These codes form the basis of the results.

**Results:**

A scientific and policy epistemic community was identified at the international and Canadian federal levels. Provincially and regionally, the community is present as an idea but its implementation varies between jurisdictions.

**Conclusion:**

The importance of economic, political and cultural factors shapes the presence and shape of the epistemic community in different jurisdictions. The community waxes and wanes but appears robust.

## Background

Canada has been recognized as a world leader in disease prevention and health promotion for some 30 years. Beginning with the Lalonde report [1], *A New Perspective on the Health of Canadians*, health services have been seen as only one of the influences on health status. The importance of addressing such determinants of health as lifestyle and environment has thus long been recognized. These ideas were expanded with the development of the Ottawa Charter for Health Promotion [2]. The Charter emphasized the need to strengthen health services by reducing inequities and influencing the determinants of health as opposed to ad hoc health promotion strategies. We argue that the Charter's overarching vision helped to create an *epistemic *or knowledge community for health promotion at international and Canadian national levels.

This paper uses the notion of epistemic community to guide analysis of linkages and gaps between health promotion policy commitments and practice at multiple health system levels in Canada in the area of heart health promotion and chronic disease prevention. Our multiple case study shows that while elements of an epistemic community can be found at the provincial level, health promotion practice in Canada remains largely a series of ad hoc strategies to reduce risk factors for disease at the local level. Furthermore, we suggest that this epistemic community, at the level of policy practice, waxes and wanes depending on the varying economic and political context.

### The idea of epistemic community

An epistemic community may be seen as a network of knowledge-based experts or groups with an authoritative claim to policy relevant knowledge within the domain of their expertise. Its members hold a common set of causal beliefs and shared notions of valid knowledge based on internally defined criteria for evaluation, common policy projects and shared normative commitments to a special research agenda [3]. In fact, the existence of such validated knowledge can be used to telling effect against the epistemic community's opponents (see [4]). Thus the importance of the existence of such communities is that they can establish and elucidate cause and effect relationships, help formulate policies and the implications of specific courses of action (see also [5]), and shed light on complex linkages and chains of events (see [6]). They can also provide channels for new ideas to circulate from societies to governments and from country to country, especially in conditions of information asymmetry between decision-makers and experts (see [7,3]).

Haas [8] demonstrates the utility of an epistemic community with respect to the barring of chlorofluorocarbons (CFCs) in which it played a key role in producing and gathering scientific information, forming a consensus with respect to the available scientific evidence, disseminating that evidence to government and corporate decision-makers. In this example the network of the community was international but its local impact varied depending on its local strength and specific national policies and configurations of interest.

Epistemic communities are not monolithic. In their study of NGOs and climate change, Gough and Shackley [4] argue that there may be differences with respect to problem construction, core beliefs and preferred responses. King [9] in his use of epistemic communities for understanding central bank reform in the U.K. makes a similar point concerning internal differences. He argues that the epistemic consensus on the need for reform and a clear roadmap for policies are necessary but insufficient conditions for institutional reform; political interest and public support as well as favourable international conditions were also important. Yet there is no doubt that an epistemic community is influential, even in poor resource and communications conditions as Karlsson [10] shows in his study of controlling communicable diseases in the Baltic Sea region and as described by Dahan et al. [11] in their study of the role of multinational corporations in transnational policy arenas.

So what may we take from the epistemic community literature for our investigation of heart health promotion policy and practice? Through their work on a commonly acknowledged subset of knowledge issues, epistemic communities create both an evidence base and a framework for policy action (i.e., a road-map). Thus an understood procedural authority is laid out by the community, which conveys the idea of progress towards the cognitive goal set by the community, usually their commitment to enhance a particular set of knowledge or actions in a particular area (e.g., effectiveness of strategies to promote health) (see [12]). To be influential, the knowledge must be codified and made explicit (see [13]). As we now turn to the development of the heart health promotion community, we in fact use its 'codebooks' or policy statements to identify the road-maps and courses of action. As we shall see, in our multiple case study scientists and policy-makers are both part of the epistemic community.

## Methods

Our research is part of a larger research program, the Canadian Heart Health Dissemination Project (CHHDP), that was designed to investigate the learnings of the provincial projects involved in the Canadian Heart Health Initiative- Dissemination Phase (CHHI) [14]. We undertook a policy analysis of national health policy and five completed provincial projects involved in the CHHI Dissemination Phase. The five provinces were Ontario (ON), Saskatchewan (SK), Manitoba (MB), Newfoundland and Labrador (NL) and Prince Edward Island (PEI). The heart health projects in these provinces focused on building capacity and disseminating heart health promotion innovations. They targeted a diverse set of organizations (public health units, health districts/regional health authorities, community committees, coalitions) and occurred in varied geographic and provincial health system contexts. Other provinces were not included because they were not part of CHHDP (New Brunswick, Nova Scotia) or adopted different approaches to heart health promotion because of financial constraints or clinical foci (Alberta, British Columbia, Quebec).

An interpretive qualitative approach was used to help recognize the importance of context in understanding diverse meanings [15]. The steps in this approach included: identification and selection of objects for analysis (policy documents, key informant interviews, project reports); thematic coding and analysis; articulation of themes and meaning; and reconciling differences in meaning.

Policy documents (n = 48) were selected representing a range of content related to dissemination and capacity building, health promotion and chronic disease prevention, health system reform or state of health system reports, and health goal documents. In addition, provincial policy documents were selected from within a timeframe of five years prior to each provincial dissemination phase and throughout the dissemination phase and national documents selected based on major turning points in the Canadian health system and health promotion policy over the last 25 years. In total, eight national health policy documents from 1974 to 2002 and 32 provincial policy documents (5–8 per province) were selected for analysis. For international context, four international health promotion declarations (1978–1997) and four international heart health promotion declarations (1994–2001) also informed this analysis.

The document analysis was supplemented by key informant interviews with provincial project research members and project stakeholders (n = 12–15 per province for a total of n = 66), with ethics approval from the McMaster University Research Board. We purposefully sampled respondents in order to achieve maximum variation on a range of views using the following sample selection criteria: range of research team members (i.e., investigators, staff); individuals involved in the project for two years or more; and stakeholders from government, non-government and community agencies. Over half the interview respondents were project stakeholders, while 45% were project research members, with an average of three years of involvement in their respective projects. Together the documents and interviews not only inform on the substantive issues of heart health/chronic disease prevention policy and practice but enable an investigation of the possibility of an epistemic community. Data on the practice of local heart health promotion communities over a four to five year period were drawn from provincial project final reports/technical analyses (n = 5) that summarize data collected from surveys, documents, interviews and focus groups.

The policy documents, interview transcripts and project reports were analyzed thematically using NUD*IST qualitative software to code, search, summarize and analyze the data [16]. Analysis included searches both within and across provincial cases based on thematic frequency and patterns of similarities and differences. A subset of policy documents (n = 3), interviews (n = 10) and project reports (n = 3) were coded by two researchers showing approximately 70% agreement on detailed coding. The level of agreement obtained for higher order themes was close to 90% and code-recode dependability was 80%, indicating good coding dependability [17]. The provincial analysis summaries were validated through a member-checking process [18] where interview respondents and project representatives, as well as provincial government policy staff reviewed summary reports to determine the accuracy of our interpretations resulting in verification and clarification.

With respect to the coding of the policy documents, the following themes emerged: the framing of health system priorities and issues, contextual factors influencing each provincial health system and the development and directions of government policy initiatives for health promotion in general, and chronic disease prevention specifically. All policy documents were reviewed with respect to document purpose; key values and principles (equity, integration, quality, accessibility); conceptual meaning and action strategies related to capacity building and dissemination; health system issues that need to be addressed or proposed solutions or actions (resource shifts, system sustainability, decision-making processes, health promotion strategies); key health system players and related sectors; and contextual factors (geography and demographic shifts, national and provincial health policy shifts, socio-economic climate).

With respect to coding the interviews, a question checklist guided interpretation and included: health promotion capacity and dissemination; interventions and changes related to capacity building and implementation; research activities; provincial context; and facilitators and barriers. The following codes emerged and included health system reform, health policy shifts, champions, funding changes or levels, geographic and demographic characteristics of provinces, history of previous heart health project phases, socio-economic climate, and political climate.

Despite this large dataset from CHHDP and international and provincial documents, limitations to our study must be noted. First those documents analyzed focus on health promotion in the context of chronic disease prevention. Our study cannot provide, therefore, an exhaustive study of epistemic communities for health promotion in Canada and internationally as it relates to other public health issues such as infectious disease, injury prevention, substance use and other population-specific health promotion efforts. Second, this analysis is also limited by the fact that the interview data were collected for the primary purposes of the CHHDP's overall investigation of intervention processes, outcomes and relationship between capacity building and dissemination in chronic disease prevention practice. Thus, we were not able to ask key informants about their perspectives on the concept of epistemic community in general and the development of such communities in Canada and internationally.

## Results

### An international epistemic community of health promotion

Internationally, a well-integrated community to support health promotion and chronic disease prevention has been developing for 30 to 35 years. Its road-map is codified at conferences at which declarations supporting science and/or advocating policy are signed. The initial charge may be seen in the Alma Ata declarations in 1978 supporting health for all in which protection and promotion were given prominent roles [19]. Eight years later, in response to and "legitimizing" [20] a growing expectation for a new global public health movement, the Ottawa Charter for Health Promotion [2] was adopted. The power of the Charter came from its widely adopted definition of health promotion: "Health promotion is the process of enabling people to increase control over, and to improve their health" [2:1]. It set a standard and provided a code for health promotion further articulated and confirmed by the Jakarta Declaration [21]. Key elements of the community – public health approach, integration, partnership and education/awareness – found in the policy advocacy documents and discourse, reinforcing the cohesiveness and strength of the health promotion epistemic community.

If the Ottawa and Jakarta Charters concern health promotion in general, heart health research and joint science-policy meetings have also confirmed and reinforced the community as an epistemic one. A significant impetus for the examination of heart disease at the population level has been the Framingham Heart Study begun in 1948, which through its research has helped identify and coalesce attention around the major risk factors (smoking, high blood pressure, overweight, physical inactivity and elevated cholesterol) [22-24]. In response to the growing body of research highlighting the burden of heart disease, the Victoria Declaration [25] put forward that much of this burden is largely preventable and established agendas for governments, international health agencies, social and economic development organizations, the scientific community, health coalitions and the private sector. In many respects, the same groups of scientists and policy makers have met on a regular basis to confirm and slightly redesign the road-map [26-28]

With each declaration from these meetings, the heart health promotion community has solidified. The messages of all the declarations are similar: prevention approaches are effective at building capacity for health promotion and subsequently reducing the rate of CVD globally. The consistent attributes and members of the community also contributed to strength at the level of ideas. Despite mixed scientific evidence of intervention effectiveness [29-35], health promotion and chronic disease prevention advocates and scientists may be seen as an epistemic community that exists with respect to courses of action commonly established to intervene in risk factor reduction. Indeed some suggest that the key outcomes of primary prevention efforts are not necessarily risk factor changes but participation rates, appearance of champions, program options, environmental changes and generation of community resources and actions (see [31,33]) The international health policy environment has developed and sustained a strong emphasis on bridging international, national, and local policy making, specifically in terms of legitimizing the broad based approach to health and overcoming barriers to change. The Declarations – the core of the advocacy component and the epistemic community – reinforce the knowledge road-map by using different lenses to point to similar strategies (as those found in the community interventions and supported by science). A cohesive and strong epistemic community in health promotion emerges at the international level. Is it present in Canada as well? And furthermore, at the level of practice?

### An epistemic community of health promotion in Canada

An epistemic community for heart health promotion in Canada is evident with strong scientific and policy advocacy roots. This is no surprise as Canadians are well-represented and strong contributors at the international level. Viewed from the present, the knowledge road-map or code book seems to be in place and there appears to be solidity to the community's existence which stems from consensus at the international and national levels around the burden of disease and the course of action to reduce risk factors and prevent disease. Elevated population levels of chronic disease and related risk factors are also well documented for Canada [36-38]. There is then agreement with respect to the scientific base of understanding the modifiable 'causes' of major chronic diseases.

The national policy impetus for health promotion in Canada began in 1974 with the release of the Lalonde report, *A New Perspective on the Health of Canadians*. This marked the beginning of the fundamental shifts in the way health professionals and government thought about chronic disease prevention and health promotion. An understanding of health promotion was later broadened beyond lifestyle factors to include environmental determinants in 1986 through the Epp Report [39], *Achieving Health for All*. Through the international consensus of the Ottawa Charter – a significant tipping point for health promotion to be seen as important and for epistemic community development – a legitimate, broad-based interpretation of health promotion for Canada was offered [20], never before articulated in national health policy. Several main prevention strategies were outlined, namely a balance of measures for the general population and high-risk groups; integrated action for improving dietary habits, eradicating tobacco use, increasing physical activity and alleviating harmful psychosocial factors; and the development of healthy public policy and the creation of supportive environments through multi-sectoral action (see [40,41]). In 1987, Health Canada formally promoted heart health [42], to help public health professionals, health agencies and community groups plan their own approaches to programs to address heart health inequalities based on socio-economic status. In fact Health Canada became not only the funder of health promotion initiatives but also a developer of ideas and strategies with many of its personnel becoming key members of the strengthening epistemic community.

But the early 1990s marked a period of fiscal constraint for the federal government. Indeed "a dramatic move in inter-provincial relations was made when the federal government introduced the Canadian Health and Social Transfer (CHST) in the 1994 budget" [20:27]. This single block funding transfer to provinces for health, social and education programs coincided with budget cutbacks. As a result, there was an increasing emphasis on (quantitative) evidence to deal with the competition for resources. The fiscal environment privileged "scientific evaluation methodologies and more medically oriented practices" that could "demonstrate positive outcomes in the shorter term" [20:27]. Health promotion programs that may have shown positive outcomes in the long term were therefore of less interest to federal funding plans. Some programs were terminated and the practice of health promotion became more challenging.

It was in this environment of fiscal restraint and demand for evidence that the shift was made from health promotion to the population health approach within the Canadian health policy landscape [43]. Understanding of the determinants of health broadened "to include up to 12 factors focused on the individual and the social and economic environment" [20:v]. Population health was offered as the new health policy paradigm in 1994 [44].

At the fourth Canadian Conference on Health Promotion, a Declaration of Intent for research was produced to "propose broad research and development orientations in the fields of health promotion and population health – orientations which can guide the work of research teams and the allocation of resources" [45: 1]. The Declaration called for an integration and collaboration between the two approaches, particularly to facilitate inter-sectoral resource allocation. The Canadian epistemic community was thus extended to an even broader, and possibly more secure, footing in policy.

The federal government, through the Health Promotion and Population Branch attempted to also integrate the two approaches by developing a "population health promotion" model [46]. Despite concerns voicing "health promotion as unduly eclipsed by population health, particularly in a context of competition for scarce resources" [20:35], population health promotion was taken up after debate and struggle by the health promotion community to ensure its own continuity.

In 1997, the Final Report of the National Forum on Health was released. Launched by the Prime Minister of Canada the Forum was to "involve and inform Canadians and to advise the federal government on innovative ways to improve our health system and the health of Canada's people" [47: 7]. Yet there is some marginalization of the health promotion community in the Forum document. It remains strong but its significance is somewhat diminished, especially with respect to the maintenance of a quality (curative) health care system with timely access for all.

Health promotion was also less central in national health system Commission reports of the early twenty-first century. Both the Romanow Report [48] and the Kirby Report [49] regard Canada as a leader of policy development in health promotion but ascribe minimal or non-existent visibility to health promotion issues in comparison to the reports' primary focus on health care. The Romanow Report embedded health promotion within primary care reform with no details as to health promotion priorities, resources allocation or specific plans on how it could be integrated within primary care systems. While the Kirby report supported its assertion that health promotion is critical, it aimed only to identify a few short-term priorities (e.g., chronic disease prevention, public health infrastructure). Does the reduced strength of policy advocacy around health promotion have implications for health promotion practice? To answer this question we turn to the provincial level.

### An epistemic community at the provincial level? Policy action and constraints

Each province has a different social, political, and economic context for developing and implementing health promotion policy. In all provinces, perhaps because of cost constraints, health promotion is favourably framed as a possible long-term solution to rising costs. Yet most of these provincial governments turned to health reforms including devolving responsibility for all health services to newly created regional authorities with little time for health promotion. Ontario was the exception to this, maintaining a provincial role in local activity delivered by regional public health units.

Overall, in all five provinces we examined, the context of competing acute care priorities and limited funding for health promotion resulted in provincial health policies that relied on local and regional community-based health promotion initiatives. Yet there is evidence of provincial policymaker and researcher adoption 'in principle' of a population health approach: a contextualized articulation of the epistemic community. There are however differences by province. In SK inter-sectoral collaboration on determinants of health based on a population health policy framework for the province ([50] and creation of a cross-department governmental structure at provincial and regional levels to address multi-sectoral issues (the Human Services Integration Forum) was emphasized. NL integrated social and health policy through the creation of a merged provincial department and regional structures for health and community services. NL also launched a Wellness Advisory Council to develop a population health based provincial Wellness Plan [51].

The other three provinces (ON, MB, PEI) developed health promotion policies largely in terms of lifestyle and behavioural approaches to address traditional lifestyle risk factors under similar economic and political pressures. Provincial government changes in ON in the 1990s resulted in a policy flux that saw a broad 'determinants of health' perspective at the beginning of the decade [52] lose its political support in 1995 when a post-recession election brought a government committed to neo-liberal reforms and budget cuts. MB had introduced a population health framework in 1992 designed to reduce inequities, establish healthy public policies, foster health promoting behaviour, and support healthy environments [53], but ongoing fiscal restraint and a change in government in the late 1990s shifted the focus of health promotion to improving life skills and encouraging healthy choices [54]. PEI merged its provincial health and social services departments using a five-health region structure to implement a provincial health promotion framework focusing on healthy communities, environment, and public policy [55]. Ten years later however the provincial health system underwent significant restructuring resulting in a separation of Health from Social Services Ministries, eliminating Health Authorities and centralizing all health services at the provincial level with few staff and no new funding to implement their Healthy Living Strategy [56].

The common theme across provincial health policies during the mid to late 1990s centred on health system funding and resource allocation. The ways that provinces chose to deal with cost constraints differed. ON, MB and NL altered funding allocation power between regions and the provincial level, opting for coalition-based health promotion strategies, with very different levels of financial support. PEI developed a strategy designed around the assumption of provincial network and agency alliances. Saskatchewan supported multi-sector initiatives and resource pooling within a population health framework supported by the regional health authorities although with no new provincial or regional resources. All five provinces advocated for partnership approaches and community participation to support regional health promotion efforts, with the ideas of the national and international epistemic communities remaining well-articulated and expressed in similar ways. But the practice of the community varies because of provincial context – economic, political and cultural (in terms of leadership). As we will see below, the expressions of the community through health promotion and chronic disease prevention practice, while broadly similar, are different at the local and regional levels.

### Implementing the code of practice: regional health promotion capacity and implementation

In this section we examine whether or not the epistemic community's scientific knowledge and policy framing is taken up and implemented in similar ways in different places to make a difference for risk factor reduction through assessing the extent of change in regional chronic disease prevention implementation over the course of provincial projects (4–5 years in duration) of the Canadian Heart Health Initiative-Dissemination Phase. Thus are the 'code-books' used in practice and programming with attention being paid to type/breadth of programming, level or amount of programming, sustainability of programs, and use of partnerships to delivery programs.

All provinces reported increased health promotion program implementation over time, although to different degrees and in different topic areas (see table 1). ON and MB reported incremental increases in heart health activity implementation over three years. In ON there was a significant increase in the delivery of heart health related activities between 1994 and 1997, whereas in MB community committees carried out more than twice the number of initiatives in their third year than they had in their first year. In PEI there was a steady increase in the participation rate of community residents in local programs, while NL showed significant growth (three-fold increase) in the number of activities implemented by regional coalitions from 142 to 441 activities over three years. In SK, changes in implementation were observed more so in process than in volume; health districts increasingly made use of partnerships and liaisons to delegate and share the responsibility for health promotion programming and inter-sectoral work increased over time.

**Table 1:** Trend of Implementation Changes*

	**ON**	**MB**	**PEI**	**NL**	**SK**
Type/breadth of programming	+	+	+	+	+

Level of programming	+	+	+	+	NC

Sustainability of programs	No data	+	+	+	No data

Use of partnerships	+	+	+	+	+

* Note: + indicates a positive change or increase in a particular dimension.

- indicates a negative change or decrease in a particular dimension. NC-no change

The interview findings show variation in terms of observed changes in areas of program implementation across the provinces. Overall, positive changes to the type/breadth (program comprehensiveness) and level of CDP/HP programming were most common. NL respondents spoke most often of changes in comprehensiveness and increases in level of program implementation. MB and PEI respondents pointed to increased sustainability of programming through community committees and coalitions. In SK the growth of partnerships and inter-sectoral collaboration for programming were highlighted. In terms of the scope of capacity development and program implementation within provinces, ON demonstrated excellent but varied capacity and program implementation growth across all public health units supported by ongoing provincial funding for a province-wide heart health program. NL also showed province wide health promotion developments and sustained commitment through a provincial wellness strategy with modest health promotion funding for regional coalitions. The other three provinces reported some enhanced capacity, despite little or no provincial investment or technical support. Thus at the regional level, the epistemic community is evident in common ideas but its practice varies. Does this mean that the community continues or will continue to make a useful contribution to health policy? Is there a fracturing at the level of practice? And are there forces at work that may exacerbate a waning of the epistemic community?

## Discussion

From this analysis of heart health promotion from the international to the provincial levels in Canada we can see the existence of a strong epistemic community based on some 50–60 years of scientific evidence concerning chronic disease and 30–40 years of policy discourse. Current research continues to identify the risk factors (see [57-59]) that are highlighted in health promotion strategies. Furthermore those strategies themselves do not appear to have changed greatly over the last 20 years or so when community involvement and healthy public policy development were added to complement individual clinical and behavioural interventions. There is therefore an epistemic consensus on risk factors which are portrayed in cause and effect terms. This consensus is strengthened by policy advocacy. International, national, and provincial levels all identify healthy public policy and community-level health promotion as priorities for enhancing health.

On the basis of our analysis 'epistemic communities' may be said to exist in health promotion at multiple system levels in Canada and is a useful analytic tool for understanding the progress and impediments in health promotion policy-practice interactions (see [60] for an alternative view). For heart health promotion and chronic disease prevention we do not see schisms or differences with respect to code, rule book, road map or policy discourse. Where there are differences is in implementation, i.e. a gap between national and provincial policy and local practice. This partly reflects the shift from international to national, provincial and regional levels in Canada and the importance of system context and resource investment in shaping implementation in particular places.

## Conclusion

Thus across national and provincial health policy in Canada over the last 25 years, several common themes resonate including funding constraints, the tension between health promotion and acute care/medicine, and jurisdictional issues between different levels of government. These in turn shape the nature and practice, but not beliefs of, the epistemic community. Furthermore public health systems in Canada have become fragmented and stressed [61]. heart health promotion and chronic disease prevention practice are in part shaped by a lack of infrastructure and capacity, leading to more volunteer-dependent and coalition action. While provinces remain advocates for health promotion and chronic disease prevention they have rarely provided sufficient funds to strengthen its capacity. As there has not been significant and sustained provincial financial investment, regional initiatives, based on community action, resource sharing and volunteer time have determined the nature and scope of implementation.

In the last few years there have been further health policy developments in Canada with a joint federal-provincial-territorial Accord on Health Care Renewal [62] which committed new investments for health care in primary care reform, home care, drug coverage, diagnostic/medical equipment, health professional recruitment/training, a patient safety strategy, a national immunization strategy, and a healthy living strategy. The Canadian government also invested $300 million (Canadian) over five years into an Integrated Strategy on Healthy Living and Chronic Disease to coordinate policies and develop specific disease strategies through inter-sectoral action in order to address obesity, physical fitness and public and environmental health needs to decrease health status differences.

Has this reinforced the epistemic community? Or has it been altered – to such an extent that the tension between health promotion ideas and practice is concerned? The Senate of Canada [63] remains a committed advocate, arguing for a federal population health policy to reduce health inequalities and improve health status. Yet the emphasis on chronic disease prevention and the strategy used to move it forward may work against an integrated health promotion community. Resources are increasingly used to target specific diseases – cancer, diabetes, heart disease – with competition and silos between disease-based groups. But much will still depend on the reactions and available resources of the provinces – the jurisdictional level responsible for health and health care in Canada- to these changes. Focusing on healthy living and chronic disease prevention may universalize the code. It may also compartmentalize it if focused on disease-specific outcomes. This code may be further weakened by the voracious appetite of acute care for resources and the public's demand for this sector's protection from financial problems.

It is possible that a way forward for strengthened health promotion implementation is to unite the multiple points of activity and support for population and public health. In this context, health promotion will require inspired champions, a confluence of public and political will for population health promotion, a collaborative federal-provincial process and commitment to concrete and integrated, multi-sector action plans. But the magic bullet remains elusive and unless appropriate policy levers are found to secure political and financial commitment this story of epistemic community development in health promotion will remain a cautionary tale of expert scientific knowledge and public health professional commitment outpacing this community's ability to support full implementation of policy practices that enhance population health.

## Competing interests

The authors declare that they have no competing interests.

## Authors' contributions

JE was responsible for developing the framework of analysis and interpretation for this paper. He was also responsible for drafting the manuscript. KR was responsible for data collection and interpretation. She added to the draft manuscript. SE was responsible for initiating the research program on which this manuscript is based. She also contributed to the draft manuscript.

## Pre-publication history

The pre-publication history for this paper can be accessed here:


